# Expression of Concern: Digital boundaries: A study on WeChat parent-child relationships among Chinese college students and their association with family factors

**DOI:** 10.1371/journal.pone.0349077

**Published:** 2026-05-11

**Authors:** The PLOS One Editors

The *PLOS One* Editors issue this Expression of Concern due to concerns about the article’s [1] compliance with the PLOS Authorship policy. Readers are advised to interpret the article with caution.
